# Heat Treatment Effect on the Phase Composition of the Silica Electrochemical Coating and the Carbon Fiber Strength

**DOI:** 10.3390/ma14185209

**Published:** 2021-09-10

**Authors:** Sergei Galyshev, Evgeniya Postnova, Olga Shakhlevich, Dmitrii Agarkov, Ekaterina Agarkova, Alexey Nekrasov, Rais Mozhchil

**Affiliations:** 1 Osipyan Institute of Solid State Physics RAS, 142432 Chernogolovka, Russia; postnova@issp.ac.ru (E.P.); olga_shh@mail.ru (O.S.); agarkov@issp.ac.ru (D.A.); stepanova.ea@issp.ac.ru (E.A.); mr_mozhchil@mail.ru (R.M.); 2 Moscow Institute of Physics and Technology, Instituskiy per. 9, Dolgoprudniy, 141701 Moscow, Russia; 3 Institute of Experimental Mineralogy, Russian Academy of Sciences, 142432 Chernogolovka, Moscow Region, Russia; alex@iem.ac.ru

**Keywords:** carbon fiber coating, electrochemical sol-gel coating, silica coating, carbon fiber

## Abstract

This work is devoted to the study of the chemical and phase composition of a carbon fiber coating obtained by the electrochemical sol-gel method. The experimental data obtained using several independent complementary methods, including X-ray phase analysis, thermogravimetric and differential thermal analysis, scanning electron microscopy and elemental analysis, and X-ray photoelectron spectroscopy, are in good agreement with each other. It was found that the resulting coating consists of amorphous silicon oxide and crystalline potassium carbonate. Heating above 870 °C leads to the crystallization of cristobalite from amorphous silicon dioxide. At a temperature of about 870 °C, the coating acquires a smooth surface, and heating above 1170 °C leads to its destruction. Thus, the optimum temperature for the heat treatment of the coating is about 870 °C. The loss of strength of carbon fiber at each stage of coating was estimated. A full coating cycle, including thermal cleaning from the sizing, coating, and heat treatment, results in a loss of fiber strength by only 11% compared to the initial state.

## 1. Introduction

The development of carbon fiber oxide coatings is an urgent task, primarily for metal matrix composites. One of the main problems in the manufacture of metal matrix composites is the chemical reaction between the matrix and the fiber because this negatively affects the mechanical properties of the composite. The use of barrier coatings of carbon fiber allows for limiting chemical interaction, which leads to an increase in the mechanical properties of the materials mentioned [1,2,3,4,5]. From a practical viewpoint, sol-gel methods are the most promising since they do not require specialized equipment or any special conditions, such as a high temperature and/or a pressure level different from the atmospheric one. Typically, these methods consist of forming a thin layer of sol on the surface of the substrate, for example, by immersion. Further, during the drying process, a thin layer of sol, due to the evaporation of the liquid, first undergoes gelation, and then dries completely, forming a porous coating. Such a coating is usually further heat treated to eliminate porosity [6]. It works well for rigid flat substrates; however, it has a number of limitations when it comes to carbon fiber coatings. These limitations are associated with the capillary structure of the fiber that leads to the formation of bridges between the fibers in the interfiber space. Another limitation is the inability to control the coating thickness over a wide range. A possible sol-gel method that allows for removing the above-mentioned limitations may be a method that combines electrochemical deposition and sol-gel technology due to a different mechanism of the coating formation [7,8,9,10].

For silica coating, this method is as follows. The substrate, which is a cathode, and an anode, is immersed in the reaction medium, which may consist of alcohol, water, salt, acid, and tetraethoxysilane. In the near-surface region of the substrate, a local change in the pH occurs as a result of an electrochemical reaction of hydrogen reduction from water. As a consequence, the local change in the pH leads to gelation in the specified region, while the main volume of the reaction medium remains in the form of a liquid solution or sol. Next, flushing is carried out, which is aimed at removing the excess liquid. After that, a thin layer of silica gel on the surface of the substrate is dried and possibly heat treated. In contrast to the sol-gel immersion method, the excess liquid is removed at the washing stage, which avoids the formation of bridges. In addition, the thickness of the coating depends directly on the deposition time and the current density.

In [11], most recently the possibility of oxide coating deposition on carbon fiber by the electrochemical sol-gel method has been shown for the first time. The mentioned work considered the effect of most of the process parameters on the structure and thickness of the coating. However, the structure, chemical and phase composition of the coating and its effect on the fiber strength were not studied. In addition, sol-gel coatings usually involve final heat treatment. The lack of information on the coating composition, the heat treatment effect, and the effect of the deposition process on the fiber strength hold back on the practical application of the electrochemical sol-gel method in the manufacture of composites with a metal matrix and carbon fiber. The main aim of the present work is to remove this obstacle.

## 2. Materials and Methods

A continuous yarn of UMT40-3K-EP carbon fiber (manufactured by UMATEX Group, Moscow, Russia) was used as a substrate. Before deposition, it was thermally purified from the sizing by annealing in vacuum at 400 °C for 15 m. Additional surface treatment of the fiber was not carried out. The silica electrochemical sol-gel coating was deposited according to the method described in [11]. Table 1 shows the parameters of the reaction medium and the mode of coating. An average coating thickness was measured from at least five SEM images of not abundant defects, similarly to [11]. It was about 1 ± 0.1 μm.

### 2.1. Heat Treatment

Heat treatment of the coated fiber yarn was carried out in a NIKIMP-2M indirect resistance furnace (NIKIMP, Moscow, USSR) in an argon atmosphere. Heating and cooling were carried out at a rate of 5°K/min. Heat treatment included heating to temperatures of 570, 870, and 1170 °C, holding for 5 m, and subsequent cooling. The indicated temperatures were recorded directly on the sample during heat treatment by a C-A thermocouple.

### 2.2. X-ray Phase Analysis

X-ray diffraction patterns were taken on a Siemens D500 diffractometer (Siemens AG, Munich, Germany) using CuKα1 radiation in the angular range of 10°–60° with a step of 0.02°.

### 2.3. Thermogravimetric (TGA) and Differential Thermal Analysis (DTA)

The TGA sample was a coated carbon fiber yarn. TGA and DTA were carried out on a Setaram Setsys Evolution 16/18 synchronous thermal analyzer in a ceramic crucible. The heating rate was 5°K/min. To increase the intensity of thermal effects and change the mass of the coating material, the base curves were recorded for a crucible with pre-annealed uncoated carbon fiber.

### 2.4. Scanning Electron Microscopy and Elemental Analysis

The samples for scanning electron microscopy and elemental analysis were prepared by spreading the fiber over the surface of a conductive tape, similarly to [11]. The analysis was performed directly from the fiber coating surface. The images were obtained using a SUPRA 50 VP high-resolution scanning electron microscope (Zeiss AG, Oberkochen, Germany) in the mode of secondary electrons with an accelerating voltage of 10 kV at magnifications up to ×50,000.

Studies to determine the elemental composition of the coating were carried out on a Tescan Vega TS5130MM digital scanning microscope equipped with an INCA Energy 450 energy dispersive spectrometer with an INCA PentaFET × 3 semiconductor Si (Li) detector (Oxford Instruments, Oxford, UK). A carbon layer with a thickness of no more than 15 nm was deposited onto the samples before the studies. The results below are the arithmetic mean of the elemental abundance calculated over at least 10 points.

An X-ray spectral local analysis was carried out at an accelerating voltage of 10 kV at a current of an electron probe on a vanadium (V) standard, used as an element for quantitative optimization of the analysis, being 200 pA. The size of the electron probe was 200 nm. The live time of accumulation of each spectrum was 100 s. The analysis results were calculated using INCA Suite version 4.15 from The Microanalysis Suite Issue 18 d + SP3 software package (Oxford Instruments, Oxford, UK). The analysis used the standards (comparison samples of the chemical composition) shown in Table 2.

### 2.5. X-ray Photoelectron Spectroscopy

X-ray photoelectron spectroscopy provides information on the elemental and valence state of the substance under study, which is especially convenient when investigating complex structures.

The studies of the electronic structure of the coating before and after heat treatment were carried out on a KRATOS AXIS ULTRA DLD photoelectron spectrometer with a spherical sector analyzer (Kratos Analytical Ltd., Manchester, UK), with the possibility of heating the sample and ion guns, ultraviolet and X-ray sources. The experiments were carried out in an ultrahigh vacuum at 5 × 10^−10^–3 × 10^−9^ Torr, using monochromatized AlK_α_ radiation of 1486.6 eV (energy resolution of 0.48 eV, binding energies were calibrated according to the Ag 3d5/2 line).

### 2.6. Fiber Strength

To determine the effect of thermal cleaning from the sizing, the coating process, and heat treatment on the strength of carbon fiber the samples were made according to the following procedure. The carbon fiber yarn was passed through a mixture of ED20-1 epoxy resin (NPK SINTEK LLC, Nizhny Novgorod, Russia) and a hardener in the ratio recommended by the manufacturer. To ensure complete impregnation of the yarn, the epoxy composition was sonicated. The impregnated thread was wound on the frame, after which the frame with the thread was kept for 72 h under normal conditions until the epoxy composition was completely cured. Using the described technique, four types of samples were obtained: reinforced with fiber in the initial state, fiber after thermal cleaning from the sizing, reinforced with coated fiber without heat treatment, and reinforced with coated fiber after heat treatment at 870 °C. The geometric shape of all the samples was a rod with an oval cross-section. The average value of the axes was about 1 and 1.2 mm, respectively.

The samples obtained were tested using a UTS-110M-5U universal testing machine (Testsystems^TM^, Ivanovo, Russia) for three-point bending strength. The strength value was calculated using the formula σ = (8FL)/(π ab^2^), where “F” is the load preceding fracture, “a” is the major axis of the ellipse in the cross-section of the specimen, “b” is the minor axis of the ellipse in the cross-section of the specimen, “L” is the distance between the supports.

To evaluate the coating cycle effect on the fiber strength at each stage, the effective strength σ_eff_ of the fiber for all sample types was calculated from the rule of mixtures. For this purpose, the flexural strength of the epoxy matrix was determined experimentally and was 129 MPa, and the initial strength of the fiber was taken from the datasheet and was σ_f_ = 4000 MPa.

## 3. Results

### 3.1. X-ray Phase Analysis

**Figure 1:**
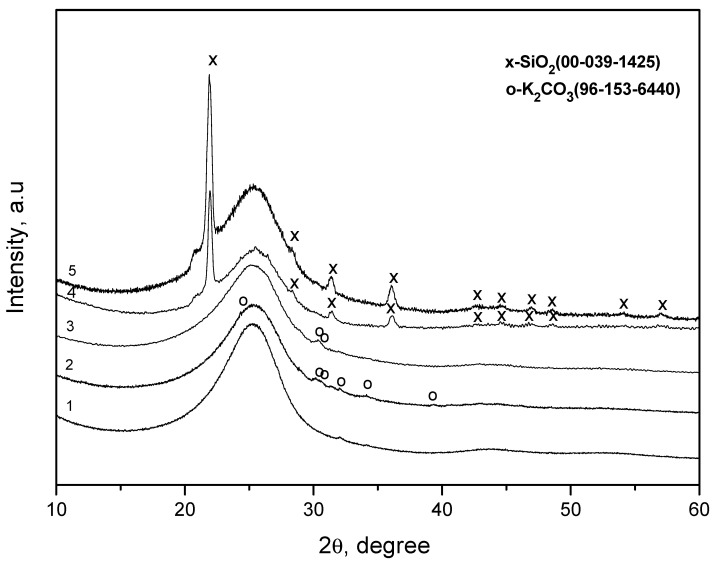
X-ray diffraction patterns of the carbon fiber surface: (**1**) uncoated; (**2**) coated before heat treatment and after heat treatment at: (**3**) 570, (**4**) 870, and (**5**) 1170 °C.

Figure 1 shows the X-ray spectra of the carbon fiber surface with a coating before and after heat treatment.

The X-ray diffraction pattern of the uncoated fiber looks characteristic of carbon fiber, and it shows a large halo corresponding to amorphous carbon. In the X-ray diffraction pattern of the coated fiber before heat treatment, in addition to the amorphous halo, peaks of low intensity are discernible that correspond to the K_2_CO_3_ compound. These peaks persist in all diffraction patterns of the coated fiber after heating. In the X-ray diffraction pattern of the coated fiber after heating to 870 °C, peaks appear characteristic of silicon dioxide in the form of cristobalite. Heating to 1170 °C leads to an increase in the intensity of cristobalite peaks.

### 3.2. Thermogravimetric and Differential Thermal Analysis

**Figure 2:**
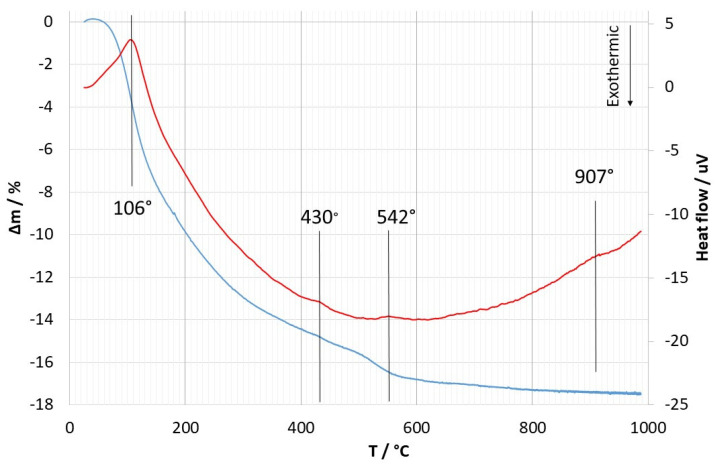
TGA and DTA curves of the coating material.

Figure 2 shows the results of TGA and DTA of the coating material. At least four endothermic peaks can be distinguished at 106, 430, 542, and 907 °C in the DTA curve. At the same time, heating to about 900 °C is accompanied by a constant decrease in the mass of the sample with one inflection in the TGA at 542 °C. When heated above 907 °C, the weight loss stops.

### 3.3. Scanning Electron Microscopy

**Figure 3:**
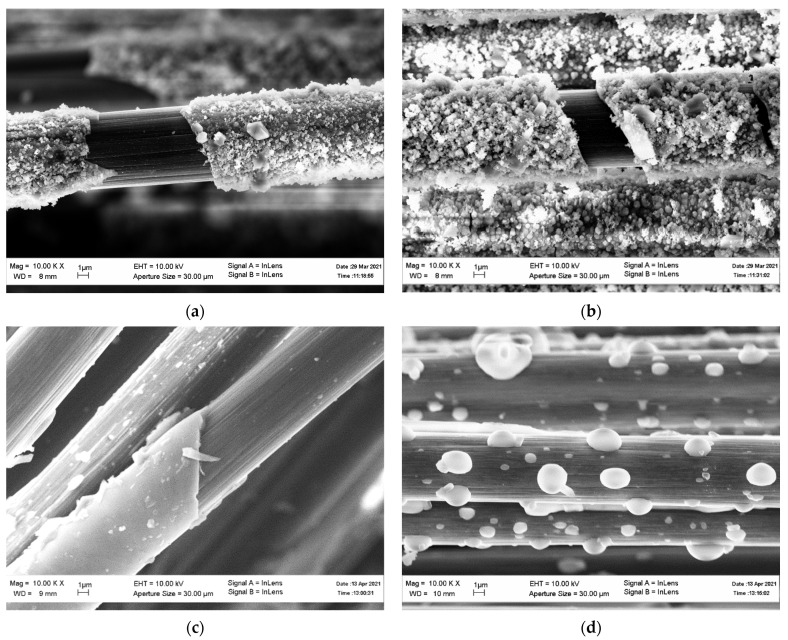
Coated carbon fiber in its original state (**a**) and after heating to (**b**) 570, (**c**) 870, and (**d**) 1170 °C.

Figure 3 shows the SEM images of coated carbon fiber in the initial state and after heat treatment. Heating up to 570 °C does not lead to external changes in the coating. As in the initial state, it looks rough and consists of rounded particles. When heated to 870 °C, the coating becomes smoother. Most probably, this is due to the mass transfer of the coating material, the driving force of which is the minimization of the surface area. Heating to 1170 °C leads to the destruction of the coating, as a result of which large, rounded particles in the form of separate drops remain on the fiber surface. This also confirms the assumption about the process of minimizing the surface area of the coating material.

### 3.4. Elemental SEM Analysis

The results of the elemental analysis of the coating material, normalized to carbon, are shown in Table 3. The column “O calc” shows oxygen content calculated on the assumption that all silicon and potassium atoms are bonded to oxygen in the forms SiO_2_ and K2CO_3_, respectively.

Note that the difference between the calculated oxygen content and the determined one is within the error of the latter. Nevertheless, in the state without heat treatment, the measured oxygen content significantly exceeds the calculated stoichiometric one (15%). As the heat treatment temperature increases, this oxygen excess decreases to 5%.

### 3.5. X-ray Photoelectron Spectroscopy (XPS)

The results of the XPS elemental analysis of the coating material, normalized to carbon, are provided in Table 4. The column “O calc” shows oxygen content calculated on the assumption that all silicon and potassium atoms are bound to oxygen in the forms SiO_2_ and K_2_CO_3_, respectively.

In contrast to the SEM elemental analysis data, a lack of oxygen is observed in the coating without heat treatment compared to the calculated one. At the same time, after heating to 1170 °C, the mentioned difference almost disappears.

**Figure 4:**
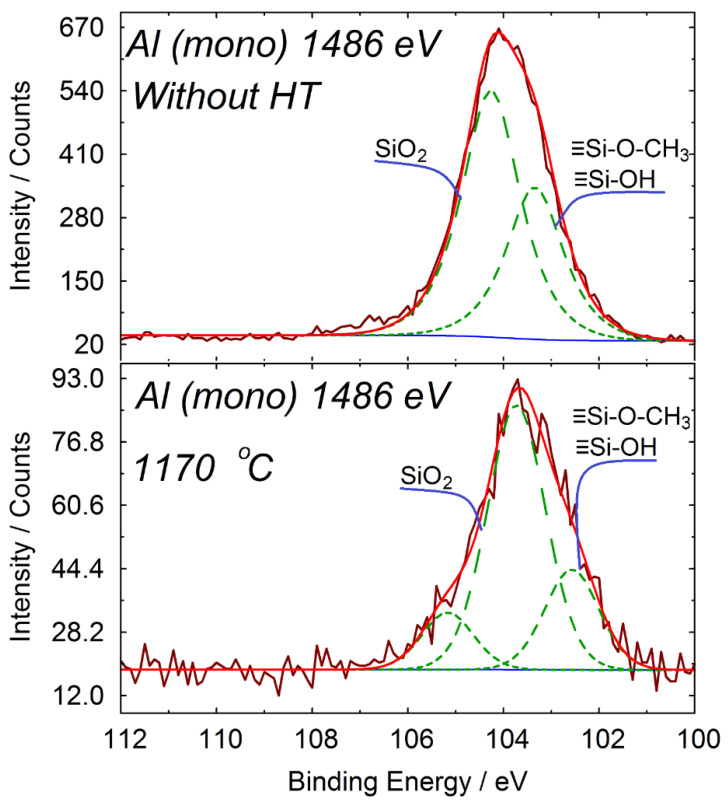
Decomposition of silicon spectrum before heat treatment and after heat treatment at 1170 °C.

The analysis of the silicon spectra (Figure 4) showed that before heat treatment, silicon is present in three states, in the form of dioxide, and in the form of silanol and ethoxy groups.

After heating to 1170 °C, the intensity of the peak corresponding to the SiO_2_ state increases compared to the spectrum before heat treatment. This means that the amount of silanol and ethoxy groups decreases during heating.

### 3.6. Fiber Strength

The results of determining the effective fiber strength in various states are presented in Table 5.

The experiment showed that the thermal cleaning of the fiber has little effect on its effective strength (−2%). The subsequent coating deposition results in a 7% decrease in the fiber effective strength. Annealing of the coating at 870 °C for 5 m reduces the effective strength of the fiber by another 4%. The total loss of fiber strength compared to the initial state is 11%.

## 4. Discussion

### 4.1. X-ray Phase Analysis

The results of XRD indicate that, before heat treatment, the coating consists of amorphous silicon oxide and a small amount of crystalline potassium carbonate. Heating to about 570 °C does not lead to significant changes in the crystalline structure of the coating. At 870 °C, a part of the amorphous silicon oxide crystallizes into cristobalite. At 1170 °C, the fraction of cristobalite increases.

Usually, peaks in the X-ray diffraction patterns of amorphous silicon oxide appear after annealing at temperatures well above 870 °C, for example, at 1200 °C [12], or at several tens of h, for example, 32 h at 950 °C [13]. The observed “low-temperature” crystallization of cristobalite can most likely be explained by the presence of an impurity of potassium carbonate. So, for example, at 8% impurity of magnesium, aluminum, and iron oxides, the first peaks in the diffraction patterns appear at 800 °C [14]. The presence of sodium ions (about 3%) reduces this temperature to 700 °C [15].

### 4.2. Thermogravimetric and Differential Thermal Analysis

The first endothermic peak in the DTA curve is observed at 106 °C; it can be associated with the loss of physically adsorbed water [13]. It corresponds to the largest inflection in the TGA curve. The next endothermic peak is observed at 430 °C; based on the XRD result, most possibly, it can be associated with the transition of potassium carbonate to the hexagonal modification [16]. The intensity of this peak is low, which corresponds to the low content of potassium carbonate and is consistent with other data. The endothermic peak at 542 °C is most likely associated with the removal of ethoxy groups, which is typical of silica synthesized by the sol-gel method [17]. It corresponds to a noticeable inflection in the TGA curve, indicating a weight loss. The endothermic peak at 907 °C corresponds to the melting of potassium carbonate and is not associated with a weight loss [18]. Note that in the temperature range from 542 to 907 °C, a slow weight loss occurs that is most likely due to the loss of chemically bound water in the form of silanol groups, which is, like a peak at 542 °C, typical of silica synthesized by the sol-gel method [19].

Note that all peaks are endothermic, which is most likely associated with the absence of oxidation of organic residues since the curves were obtained in an argon atmosphere.

### 4.3. Scanning Electron Microscopy

According to the XRD data, before heat treatment, the coating material consists of amorphous silicon oxide and a small amount of crystalline potassium carbonate. Heating above 870 °C leads to the crystallization of amorphous silicon oxide to cristobalite. In the specified temperature range, a process of mass transfer occurs in the coating material, most probably due to a decrease in the surface energy. At the same time, at a heat treatment temperature of 870 °C, the coating acquires a smooth surface. Heating to 1170 °C leads to the destruction of the coating, the coating material coagulates as droplets on the fiber surface, indicating an active mass transfer.

The results obtained agree with the results from [14,15], where it is shown that, during heating, silicon oxide particles are merged to each other [14], and the total surface area is reduced [15]. However, it should be noted that the degree of coagulation observed in this work is much higher than that in [14]. This is most likely due to the presence of potassium carbonate, the presence of which accelerates diffusion processes.

Note that all the experimental data obtained using several independent methods are in good agreement with each other. From a practical point of view, it can be concluded that the optimum temperature for the annealing of the coating is in the range from 570 to 870 °C.

### 4.4. Elemental SEM Analysis and XPS

Based on the data of SEM and XPS elemental analyses, the coating material consists of silicon, potassium, oxygen, and carbon. In this case, the presence of silicon, oxygen, and carbon is quite expected, while potassium is most likely to be initially included in the coating in the form of ions from the reaction medium, which are attracted to the fiber cathode, subsequently forming carbonate.

Since the SEM elemental analysis is performed from the bulk of the material with a depth of about 1 μm, and the XPS analysis is performed from surface layers at a depth of less than 5 nm, the results obtained can be considered the elemental composition of the coating in the bulk and on its surface, respectively.

From this point of view, the measured oxygen excess (compared to the calculated) in the SEM analysis can be explained by the presence of physically adsorbed water, which is in good agreement with the DTA and TGA data and [13]. The measured oxygen deficiency (compared to the calculated one) in the XPS analysis indicates the absence of physically adsorbed water on the coating surface. Decomposition of the silicon peak in the XPS analysis indicates the presence of silanol and/or ethoxy groups on the coating surface. Therefore, in the near-surface layer, one silicon atom will have not two (like SiO_2_) but one oxygen atom, which determines the measured oxygen deficiency. Thus, the obtained results indicate that, prior to heat treatment, physically adsorbed water is present mainly in the bulk of the coating material, whereas organic residues are located predominantly on its surface.

### 4.5. Fiber Strength

From a practical point of view, the loss of the fiber strength during the coating process needs to be as small as possible. The loss of strength depends largely on the deposition method. Thus, when a titanium carbide layer with a thickness of 200 nm is formed using the molten salt method, the loss of strength reaches 60% [20]. The CVD deposition also results in quite a large loss in the fiber strength. Thus, when pyrolytic carbon was deposited in [21], the loss of strength was about 50%, and when titanium nitride was deposited in [22], it was about 40%. A lower loss of strength can be ensured by lowering the CVD temperature, so in [23], when Al_2_O_3_ was deposited, the loss of fiber strength was only 19%. An even lower loss of strength of about 17% was achieved by the authors of [1] using the sol-gel immersion method. This effect of the coating method is most likely due to the chemical interaction between the fiber and the precursors forming the coating as in [20]. This assumption is confirmed by the results mentioned in [21,22,23] showing a decrease in the loss of strength with a decreasing temperature of the deposition process. The results obtained in this work, where the loss of fiber strength was only 13%, fit well into the above logic. This is because one of the advantages of the sol-gel methods is that, usually, they do not require high temperatures and the reagents used do not interact with fiber.

## 5. Conclusions

1. The chemical and phase composition of the coating of carbon fiber obtained by electrochemical deposition from an aqueous-alcoholic solution of tetraethoxysilane has been studied. The experimental data obtained using several independent complementary methods are in good agreement with each other. It has been found that the resulting coating consists of amorphous silicon oxide and crystalline potassium carbonate. Heating above 870 °C leads to the crystallization of cristobalite from amorphous silicon dioxide. At a heat treatment temperature of 870 °C, the coating acquires a smooth surface, and heating to 1170 °C leads to its destruction.
2. In the temperature range from 870 to 1170 °C, a process of mass transfer occurs in the coating material. At a heat treatment temperature of 870 °C, the coating acquires a smooth surface. Heating to 1170 °C leads to the destruction of the coating, and the coating material coagulates as droplets on the fiber surface, indicating an active mass transfer.
3. The elemental SEM analysis and XPS results indicate that, prior to heat treatment, physically adsorbed water is present mainly in the bulk of the coating material, whereas organic residues are located predominantly on its surface.
4. A full cycle of coating, including thermal cleaning from the sizing, coating, and annealing at 870 °C for 5 m, leads to a decrease in the effective fiber strength by 13% compared to the initial state.

## Figures and Tables

**Table 1:** Parameters of the reaction medium for coating.

C_IPA_, vol. %	MR	C_salt_, g/L	pH	J, mA/cm^2^	τ, min
67	62	20	2.23	5.3	1.5

**Table 2:** Standards (comparison samples of the chemical composition) used in the elemental analysis of the coating.

Standard	Element	Chemical Composition (Mass %)
BN	N	B—47.55%, N—56.45%
Quartz (SiO_2_)	O, Si	O—53.26%, Si—46.74%
Albite	Na	O—48.5%, Na—8.38%, Al—10.48%, Si—31.79%, Ca—0.1%
Orthoclase	K	O—46.57%, Na—2.74%, Al—10.53%, Si—30.06%, K—9.46%, Ca—0.09%

**Table 3:** Results of the elemental analysis of the coating material, normalized to carbon (at.%).

Coating Condition	O calc	O	Si	K
Without HT	55.95	70.3 ± 17	22.8 ± 5.5	6.9 ± 2.8
HT 570 °C	52.85	72 ± 13.6	21.7 ± 5.5	6.3 ± 2.6
HT 870 °C	60	68.9 ± 12.6	26.7 ± 7.3	4.4 ± 3.6
HT 1170 °C	62.45	67.7 ± 11	28 ± 6	4.3 ± 2.8

**Table 4:** Results of the XPS elemental analysis of the coating material, normalized to carbon (at.%).

Coating Condition	O calc	O	Si	K
Without HT	73.22	61.6	31.24	7.16
HT 1170 °C	66.33	64.76	26.94	8.3

**Table 5:** Strength of composite rods (σ_cr_) and relative effective fiber strength (σ_eff_/σ_f_) in different states.

Fiber State	σ_cr_, MPa	σ_eff_/σ_f_, %
Initial	1683 ± 261	98
After thermal purification	1514 ± 123	96
Coating without HT	1086 ± 46	91
Coating annealed at 870 °C	1126 ± 93	87

## Data Availability

The data presented in this study are available on request from the corresponding author.
